# Time-dependency of edema-based assessment of area-at-risk in reperfused acute myocardial infarction

**DOI:** 10.1186/1532-429X-13-S1-P111

**Published:** 2011-02-02

**Authors:** Veronica LM Rundell, Xiangzhi Zhou, Avinash Kali, Ying Liu, Richard Tang, Rachel A Klein, Andreas Kumar, Rohan Dharmakumar

**Affiliations:** 1 Northwestern University, Chicago, IL, USA; 2 Laval University, Quebec City, QC, Canada

## Introduction

Relative edema volume, computed from T2-based CMR images, has been used to define the Area-At-Risk in myocardial infarction and in the determination of salvagable myocardium in patients. Following infarction, edema presents as hyperintense regions in T2-weighted CMR, however, the literature reports disparate data resulting from delayed imaging and type of MR acquisition.

## Purpose

To investigate the time course and extent of myocardial edema in reperfused myocardial infarction on the basis of T2 maps and T2-weighted STIR imaging.

## Methods

Cardiac MR was used to investigate the development and persistence of edema volume increases in the acute post-infarct period using both T_2_-weighted STIR imaging and T_2_ maps. LAD instrumented canines (n=9) underwent three hours of no-flow ischemia followed by reperfusion and were studied [using Siemens 1.5 T Espree scanner] at five time points (before and during ischemia and 2, 5, 7 and 56 days post-reperfusion). Scan parameters for T_2_-STIR acquisitions were: TE=64ms, TR=2-3 R-R intervals, resolution=0.9x0.9x8.0mm^3^. T_2_ maps were computed from multiple T_2_-prepared acquisitions with different preparation times (0, 24, and 55 ms) with spatial-resolution of 1.0x1.0x8.0mm^3^. Late-enhancement imaging confirmed LAD infarction. Areas of interest were determined as follows: For T2 maps - region with T_2_ values greater than 2SD from remote territories; and for T_2_-STIR images- regions exceeding 2SD of the signal intensity of the remote regions. Edema volume was computed as highlighted areas multiplied by imaging slice thickness. Percent volume of edema was computed relative to total myocardial volume. Results are reported as mean ± SEM for each time point.

## Results

**Figure 1:**
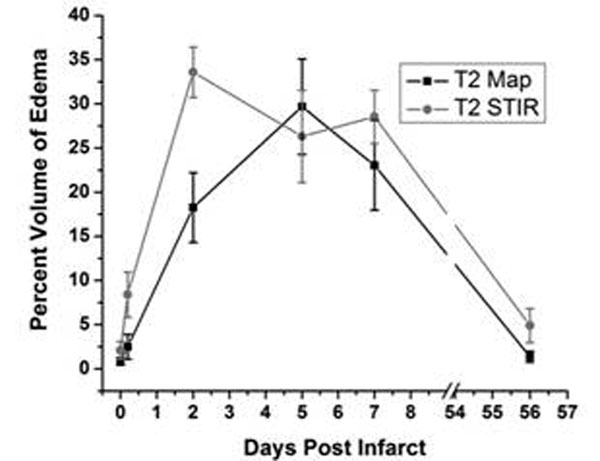
Time evolution of edema volume in a canine model of ischemia-reperfusion injury measured on the basis of changes in T2 and signal intensity on T2-STIR images: Note that the percent volume of edema measured from T2 maps is generally lower than that from T2 STIR and that the edema volume is variable within the acute period of tissue injury and resolves to near baseline levels by week 8.

Percent volume of edema (Area-At-Risk) - **T_2_ Maps:** Pre-ischemia: 0.8±0.4%; Ischemia: 2.5±1.4%; Day 2: 18.3±3.9%; Day 5: 29.7±5.4%; Day7: 23.0±5.1%; and Day 56: 1.4±0.6%. **T_2_-STIR:** Pre-ischemia: 2.1±1.0%; Ischemia: 8.4±2.6%; Day 2: 33.6±2.9%; Day 5: 26.1±5.2%; Day7: 28.5±3.0%; and Day 56: 4.9±1.9%. p<0.01 by ANOVA. T_2_ maps showed a continuous rise in relative edema volume post reperfusion that peaked at day 5. Although edema volume decreased by day 7, it remained significantly elevated from pre-ischemic levels. In T_2_-STIR analysis, edema volume rose rapidly, peaking at day 2 and remaining elevated throughout the acute study period. T_2_-STIR images showed an earlier peak in edema volume compared to that determined from T_2_ maps. Both measures demonstrated regression of edema after 56 days. Figure 1.

## Conclusion

This data indicates that the time following ischemia-reperfusion injury, as well as the mode of MR interrogation, are critical variables in the determination of Area-at-Risk.

